# Targeting Ferroptosis: Pathological Mechanism and Treatment of Ischemia-Reperfusion Injury

**DOI:** 10.1155/2021/1587922

**Published:** 2021-10-28

**Authors:** Xinye Li, Ning Ma, Juping Xu, Yanchi Zhang, Pan Yang, Xin Su, Yanfeng Xing, Na An, Fan Yang, Guoxia Zhang, Lijing Zhang, Yanwei Xing

**Affiliations:** ^1^Guang'anmen Hospital, China Academy of Chinese Medical Sciences, Beijing 100053, China; ^2^Beijing University of Chinese Medicine, Beijing 100029, China; ^3^Dezhou Second People's Hospital, Dezhou 253000, China; ^4^The Second People's Hospital of Jiaozuo, Jiaozuo 454001, China; ^5^The First Affiliated Hospital, Hebei North University, Zhangjiakou 075000, China; ^6^Shanxi University of Chinese Medicine, Taiyuan 030619, China; ^7^Dongzhimen Hospital, Beijing University of Chinese Medicine, Beijing 100700, China

## Abstract

Ischemia-reperfusion (I/R) is a pathological process that occurs in many organs and diseases. Reperfusion, recovery of blood flow, and reoxygenation often lead to reperfusion injury. Drug therapy and early reperfusion therapy can reduce tissue injury and cell necrosis caused by ischemia, leading to irreversible I/R injury. Ferroptosis was clearly defined in 2012 as a newly discovered iron-dependent, peroxide-driven, nonapoptotic form of regulated cell death. Ferroptosis is considered the cause of reperfusion injury. This discovery provides new avenues for the recognition and treatment of diseases. Ferroptosis is a key factor that leads to I/R injury and organ failure. Given the important role of ferroptosis in I/R injury, there is considerable interest in the potential role of ferroptosis as a targeted treatment for a wide range of I/R injury-related diseases. Recently, substantial progress has been made in applying ferroptosis to I/R injury in various organs and diseases. The development of ferroptosis regulators is expected to provide new opportunities for the treatment of I/R injury. Herein, we analytically review the pathological mechanism and targeted treatment of ferroptosis in I/R and related diseases from the perspectives of myocardial I/R injury, cerebral I/R injury, and ischemic renal injury.

## 1. Introduction

**Figure 1:**
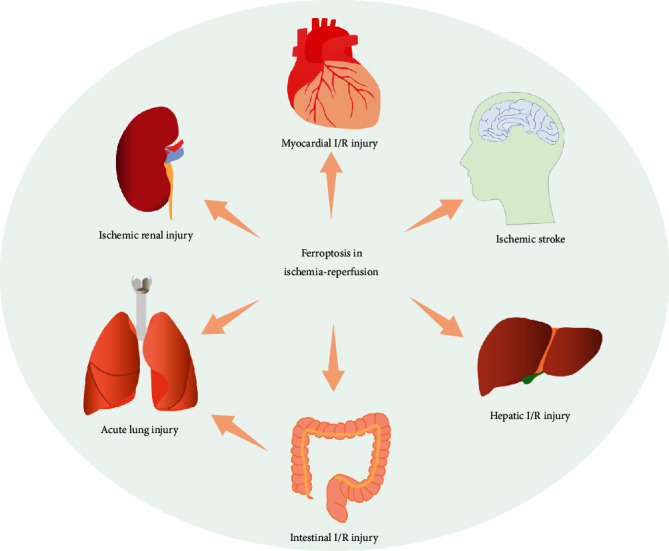
Ferroptosis plays important roles in ischemia-reperfusion (I/R) injury, including myocardial I/R injury, cerebral I/R injury, ischemic renal injury, hepatic I/R injury, intestinal I/R injury, and acute lung injury induced by intestinal I/R.

Ischemia-reperfusion (I/R) is a pathological process that occurs in numerous organs and diseases and can lead to cellular damage and death. Ischemia occurs when the blood supply to organs is restricted as a result of an embolus that blocks arterial blood supply. Ischemic events are related to a serious imbalance in cell metabolism, resulting in tissue hypoxia. In the affected ischemic area, reperfusion and restoration of blood flow and reoxygenation are frequently related to “reperfusion injury,” including excessive tissue injury and destructive inflammatory responses [1]. I/R injury is a major pathological factor in many diseases, especially after cardiac trauma. Additionally, I/R injury delays the recovery of transplanted organs and patients undergoing treatment. Pharmacological therapy and early reperfusion therapy can reduce tissue damage and cell necrosis induced by ischemia. However, these treatments can also cause irreversible I/R injuries. Cell death is the major common I/R injury characteristic in all tissues, and consequently, it is a stable pathological index of I/R injury. Ferroptosis is a newly recognized form of programmed cell death that relies on iron and reactive oxygen species (ROS) [2] and is believed to be the cause of reperfusion injury [3]. Thus, the new manner of cell death, ferroptosis, provides new avenues for understanding and treating many diseases. Research on I/R injury has been conducted in numerous organs, including the heart [4], brain [5], kidney [6], and liver [7]. Recent studies have revealed the links between ferroptosis and I/R injury (Figure 1), and ferroptosis inhibitors have successfully prevented or reduced I/R injury in various organs. Ferroptosis is a key driver of I/R injury and organ failure. Inhibiting ferroptosis may become an effective treatment strategy for related organ diseases and will help to reduce cell death during reperfusion injury. Therefore, it is worth emphasizing the importance of exploring the pathological mechanism of ferroptosis in I/R and targeted therapies. In this article, we summarize the available evidence regarding the pathological mechanism of ferroptosis, I/R injury in organs and diseases, and research progress on related therapeutic targets.

## 2. Regulation Mechanism of Ferroptosis

**Figure 2:**
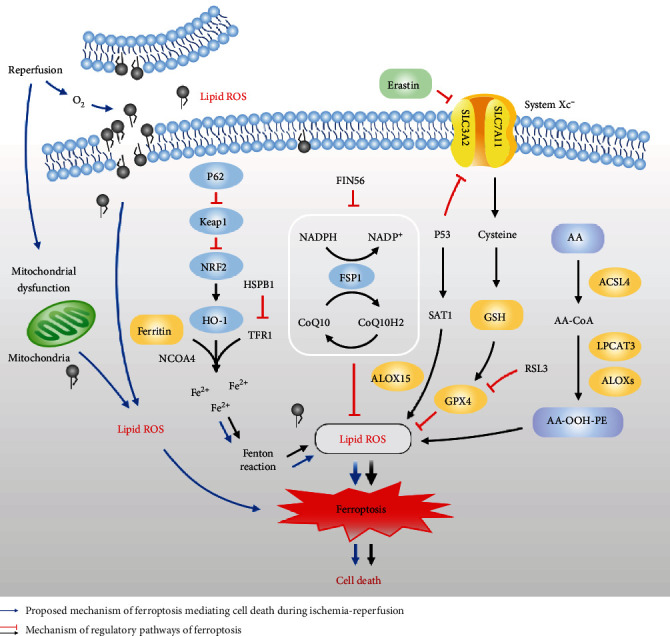
Schematic representation of the proposed mechanism of ferroptosis mediating cell death during ischemia-reperfusion and the mechanism of regulatory pathways of ferroptosis. SLC3A2: solute carrier family 3 member 2; SLC7A11: solute carrier family 7 member 11; Keap1: Kelch-like ECH-associated protein 1; NRF2: nuclear factor erythroid 2-related factor; HO-1: heme oxygenase-1; NCOA4: nuclear receptor coactivator 4; HSPB1: heat shock factor-binding protein 1; TFR1: transferrin receptor 1; FIN56: ferroptosis inducing 56; FSP1: ferroptosis suppressor protein 1; CoQ10: coenzyme Q10; RSL3: (1S, 3R)-RSL; GSH: glutathione; GPX4: glutathione peroxidase 4; ROS: reactive oxygen species; SAT1: spermidine/spermine N^1^-acetyltransferase 1; ALOX-15: arachidonate lipoxygenase 15; AA: arachidonic acid; ACSL4: acyl-CoA synthetase long-chain family 4; LPCAT3: lysophosphatidylcholine acyltransferase 3; ALOXs: arachidonate lipoxygenases; PE: phosphatidylethanolamine.

Ferroptosis is an iron-dependent, peroxidation-driven, nonapoptotic form of regulated cell death [3, 8, 9]. It is biochemically, morphologically, and genetically different from apoptosis, necrosis, and other forms of cell death [2, 3, 10, 11]. Ferroptosis was clearly defined in 2012 when it was demonstrated that erastin could inhibit the antioxidant glutathione (GSH) synthesis and trigger iron-dependent cell death, which was also nonapoptotic [2]. Multiple biological regulatory pathways work simultaneously during ferroptosis. GSH deficiency or glutathione peroxidase 4 (GPX4) inactivation causes ferroptosis [2, 12–14]. Furthermore, ferroptosis is characterized by iron-mediated excessive peroxidation of polyunsaturated fatty acids (PUFAs) [15]. The immoderate accumulation of iron-dependent lipid hydroperoxides leads to ferroptosis. The mechanisms and major regulatory factors involved in ferroptosis are shown in Figure 2.

### 2.1. Antioxidant Mechanism of Ferroptosis

System *x*_*c*_^−^ is a cystine-glutamate exchanger, which consists of heterodimers of the solute carrier family 3 member 2 (SLC3A2) and the catalytic subunit solute carrier family 7 member 11 (SLC7A11) [16, 17]. System *x*_*c*_^−^ promotes the exchange process across the plasma membrane of cystine and glutamate. Cystine is necessary for cell survival and is the predominant form of cysteine in extracellular space [18]. System *x*_*c*_^−^ imports extracellular cystine for GSH synthesis and transfers glutamate out of cells [19]. GPX4, an antioxidant enzyme that maintains cellular redox homeostasis, primarily acts as a crucial endogenous antioxidant against phospholipid peroxide, mainly using GSH as a cofactor [20]. The inhibition of system *x*_*c*_^−^ induces GSH depletion and causes the inactivation of GPX4, resulting in the accumulation of toxic lipid ROS and triggering ferroptosis [2, 14]. To maintain the function and activity of GPX4, GSH and selenium (Se) are indispensable [3, 21]. GSH also acts as a major endogenous antioxidant [22] and participates in the regeneration of GPX4 [23]. GSH is synthesized in two steps from glutamate, cysteine, and glycine under the catalysis of enzymes [17]. Erastin and (1S,3R)-RSL (RSL3) were identified as compounds that induce RAS-mutated tumor cell death [9, 24], and some proteins associated with GSH metabolism are related to ferroptosis [8]. GPX4 can act directly or indirectly by ferroptosis agonists, thereby reducing its activity [25]. RSL3 is a ferroptosis inducer that directly inhibits GPX4 by covalently targeting selenocysteine. It inhibits GPX4 activity, thereby causing intracellular accumulation of lipid peroxides and subsequent cell death [14, 26]. Erastin, another representative ferroptosis inducer, depletes cellular cysteine and reduces the biosynthesis of GSH by directly inhibiting system *x*_*c*_^−^ [13]. This leads to the loss of GPX4 activity, leading to the accumulation of lipid peroxides and eventually ferroptosis. The main target of erastin is the exchange of cystine/cystathionine with glutamate [2]. Recent evidence has shown that the ferroptosis suppressor protein 1- (FSP1-) coenzyme Q10- (CoQ10-) (nicotinamide adenine dinucleotide phosphate) NAD(P)H pathway synergistically inhibits the proliferation of lipid peroxides and ferroptosis with GPX4 and GSH, as an independent system [27, 28]. FSP1 functions as an oxidoreductase that reduces CoQ10 using NAD(P)H and produces a lipophilic radical-capture antioxidant (RTA) to prevent the production of lipid peroxides [27, 28]. Ferroptosis inducing 56 (FIN56) has been shown to cause ferroptosis by blocking this pathway [29]. The tumor suppressor protein P53 plays a role in apoptosis, necrosis, and autophagy. P53 can repress SLC7A11 gene expression, affecting the activity of GPX4 and leading to the accumulation of lipid peroxidation and an increase in ferroptosis [30, 31]. P53-SAT1- (spermidine/spermine N^1^-acetyltransferase 1-) arachidonate lipoxygenase 15 (ALOX-15) pathway also participates in the regulation of ferroptosis [32]. SAT1 acts as a transcriptional target of P53, and its activation can induce ROS-induced lipid peroxidation and ferroptosis.

### 2.2. Oxidation Mechanisms of Ferroptosis

PUFAs are oxidized to form lipid peroxides [33]. The important factors that cause ferroptosis include the accumulation of lipid peroxides [2, 3]. Oxidized arachidonic acid-containing phosphatidylethanolamine (AA-PE) acts as a signal of ferroptosis. A recent study showed that AA-OOH-PE, instead of other types of phospholipid-OOH, is the key phospholipid that induces ferroptosis [34]. Three enzymes are required for the generation of AA-OOH-PE: arachidonate lipoxygenases (ALOXs), acyl-CoA synthetase long-chain family 4 (ACSL4), and lysophosphatidylcholine acyltransferase 3 (LPCAT3) [35]. ACSL4 catalyzes PUFA (especially AA) to yield CoA-forms [36], and LPCAT3 regulates the esterification of AA-CoA into AA-PE [15]. Under the catalysis of lipoxygenase (LOXs), AA-PE can be oxidized to AA-OOH-PE and play further oxidative roles [26, 34, 37]. When the AA-OOH-PE level exceeds the limit of the reduction system, ferroptosis is induced [17, 36, 38]. In addition to lipid peroxidation, iron, a redox-active metal, is necessary for ferroptosis [39]. As the iron source of the Fenton reaction, in the labile iron pool (LIP), small amounts of iron are free and chelated. Iron released from the LIP can subsequently promote ROS accumulation through the Fenton reaction [40–42]. During ferroptosis, ROS mainly originate from the Fenton reaction. In addition, iron is a prooxidant in ferroptosis and is vital for the function of enzymes, such as lipoxygenases (LOXs) [43, 44]. Ferroptosis is also regulated by proteins involved in iron homeostasis. ShRNA-mediated iron response element binding protein 2 (IREB2) silencing has been demonstrated to reduce sensitivity to ferroptosis by modifying iron uptake, metabolism, and storage [2]. Cysteine desulfurase (NFS1) has also been shown to reduce ferroptosis in lung cancer [45]. Heat shock factor-binding protein 1 (HSPB1), a negative regulator of ferroptosis, reduces iron concentrations by inhibiting the expression of TRF1, thereby inhibiting ferroptosis [46]. Ferritin autophagy can increase the content of Fe^2+^ [47], thus promoting ferroptosis. At the same time, the ferritin receptor nuclear receptor coactivator 4 (NCOA4) mediates the autophagic degradation of ferritin, increasing free iron [48, 49]. Heme oxygenase-1 (HO-1) decomposes heme to release iron, thereby accelerating ferroptosis [50]. P62 is an autophagy receptor that can inhibit Kelch-like ECH-associated protein 1 (Keap1) directly and promotes nuclear factor erythroid 2-related factor (NRF2) activation simultaneously. An early study showed that oxidative stress induces the expression of HO-1 by activating the p62-Keap1-NRF2 pathway, thus antagonizing ferroptosis [51]. Nrf2 activates the expression of various target genes necessary to regulate ferroptosis by regulating the metabolism of GSH, lipid, iron, and mitochondrial function [51].

## 3. Pathological Mechanisms and Potential Targeted Therapy of Ferroptosis in Ischemia-Reperfusion Injury

### 3.1. Role of Ferroptosis in Ischemia-Reperfusion-Related Cell Death

Previous studies have shown that ferroptosis occurs during the reperfusion period and not during the ischemic period in I/R injury models [52]. After I/R, the endogenous mechanisms of scavenging ROS are seriously negated, and ROS cannot be effectively scavenged. Reperfusion of ischemic tissue causes excessive ROS, which mediate I/R injury [53, 54]. There is evidence that I/R injury is accompanied by several cellular events, such as reperfusion-related excessive ROS accompanied by lipid peroxidation [55] and an increase in intracellular iron concentration [56, 57]. Lipid peroxidation and ROS lead to cell injury and death. These cellular events are consistent with the manifestation of iron-dependent ferroptosis, which could be prevented by iron chelators [58]. Figure 2 shows the proposed mechanism of ferroptosis, which mediates cell death during ischemia-reperfusion. Current studies have shown that phospholipid oxidation products exist in myocardial I/R injury [59–62]. In addition to lipid peroxidation, free iron is a necessary condition for ferroptosis. Nonenzymatic production of ROS occurs when metal ions are present. There is a LIP in the cytosol, mitochondria, and lysosomes of cells [41]. The free iron in the LIP could accelerate the lipid peroxidation of saturated fatty acids in humans via the Fenton reaction [63], and iron is involved in producing ROS in mitochondria. Mitochondria of a cell are the main sites for generating ROS [64, 65], and after the injury, they undergo alterations of structure and function. During ischemia, mitochondrial dysfunction leads to excessive ROS production. Therefore, current evidence supports the involvement of ferroptosis in the progression of organ cell injury after I/R. Ferroptosis is an important mechanism that mediates cellular damage to organs and cell death during I/R.

### 3.2. Myocardial I/R Injury

#### 3.2.1. Ferroptosis in Myocardial I/R Injury

Coronary artery disease has been suggested to be the most common cardiovascular disease [66], and atherosclerosis is the main cause. Plaque rupture results in acute ischemia and myocardial infarction. A large amount of ROS is produced after reperfusion [67, 68], leading to further damage [69]. During ischemia and early reperfusion, ferritin degrades to release iron and promotes iron-mediated Fenton reaction, leading to oxidative damage and loss of cardiac function associated with I/R injury. In experimental models, inhibition of glutaminolysis, an important part of ferroptosis, was shown to reduce I/R-induced heart injury [70]. Studies have confirmed that ROS accumulation and iron content increase during I/R injury [71, 72], and iron overload is an important cause of myocardial cell injury [73]. A study by Li et al. showed that iron deposition and ROS overproduction occurred during diabetic myocardial injury, and ferroptosis was involved in the I/R injury of diabetic myocardium through the endoplasmic reticulum stress pathway [31]. These studies indicate that ferroptosis plays a key role in myocardial I/R injury. Transferrin receptor 1 (TfR1) is involved in cellular iron uptake. A novel pathway of ubiquitin-specific protease 7 (USP7)/p53/TfR1 is found in rat hearts after I/R treatment, and the upregulation of USP7 accelerates ferroptosis by activating the p53/TfR1 pathway [74]. The study has shown that ferroptosis occurs during reperfusion in rat hearts subjected to I/R [52]. With the prolongation of reperfusion time, the levels of ACSL4 protein in the cardiac tissue gradually increased, accompanied by a decrease in GPX4 levels. The exact role of ferroptosis in myocardial I/R injury remains unclear. Other studies have shown that inhibition of ferroptosis has a protective effect on myocardial I/R-induced rats [75].

#### 3.2.2. Target and Targeted Therapy of Ferroptosis in Myocardial I/R Injury

Targeting ferroptosis is considered a feasible treatment for I/R injury. The exposure of rat cardiomyocytes to simulated I/R injury can cause nonapoptotic cell death, and the addition of ferrostatin-1 during reperfusion can attenuate this effect [76]. Fang et al. [77] found that the in vivo application of ferristatin-1 and an iron chelator plays a role in reducing heart failure caused by I/R. It has been suggested that ferroptosis is related to myocardial I/R injury, and ferristatin-1 may be a feasible treatment to alleviate reperfusion injury. In addition, many studies have demonstrated that iron chelators can block ferroptosis in vitro and in vivo. Deferoxamine (DFO) is the most widely used nontoxic iron chelator that inhibits ferroptosis mediated by lipid peroxidation under various conditions [2]. Chan et al. showed that DFO administered before reperfusion by primary percutaneous coronary intervention could rapidly ameliorate oxidative stress but failed to limit infarct size [78]. In a study by Paraskevaidis et al., intravenous DFO infusion protected the myocardium from reperfusion injury and reduced lipid peroxidation during coronary artery bypass grafting surgery [79]. DFO, which binds iron, has been shown to have a protective effect on myocardial I/R [70]. Shan et al. demonstrated that Cyanidin-3-glucoside (C3G) treatment reduced oxidative stress and Fe^2+^ content, confirming that C3G effectively alleviated myocardial I/R injury by inhibiting ferroptosis both in vivo and in vitro [72]. C3G treatment could reduce myocardial infarction area, inhibit pathological damage, and inhibit ST-segment elevation. Baicalein (also termed 5,6,7-trihydroxyflavone) is a flavonoid that limits iron accumulation induced by erastin and lipid peroxidation by inhibiting GPX4 degradation and GSH consumption [80]. In addition, baicalein significantly reduced iron-induced lipid peroxidation and inhibited the expression of ALOX-15 in HT22 cells [81]. A recent study has shown that baicalin protects against myocardial I/R injury via inhibiting ACSL4-controlled ferroptosis in a rat model [82]. Ma et al. found that ubiquitin-specific peptidase 22 (USP22) inhibits cardiomyocyte death induced by ferroptosis in myocardial I/R injury via the SIRT1/p53/SLC7A11 axis, providing a novel therapeutic target for the treatment [83]. Chen et al. found that embryonic lethal-abnormal vision like protein 1 (ELAVL1) increased substantially during myocardial I/R injury. ELAVL1 knockout decreased ferroptosis, thus ameliorating I/R injury [84]. FOXC1-activated transcription mediates the increase in ELAVL1 induced by myocardial I/R, and FOXC1 plays a key role in myocardial I/R injury [84]. Inhibition of ELAVL1-mediated ferroptosis might be a new approach for the treatment of myocardial I/R injury. Oxidized phosphatidylcholine (OxPC) production increases during myocardial I/R injury and causes extensive cell death through ferroptosis [85]. Interventions targeting OxPCs may help reduce ferroptosis during I/R injury. Determining appropriate therapeutic targets is vital. Further studies are needed to establish more effective anti-ferroptosis-targeted therapies for patients with myocardial ischemia.

### 3.3. Cerebral I/R Injury

#### 3.3.1. Ferroptosis in Cerebral I/R Injury

Ischemic stroke is the main cause of destructive cerebrovascular disease and has high mortality and morbidity rates [86]. Restoring blood supply to the ischemic area as soon as possible is crucial for ischemic cerebrovascular disease treatment [87]. Early reperfusion is the most effective therapy for acute cerebral ischemia; however, the increase in ROS and inflammation, and other phenomena in tissues after reperfusion aggravates brain tissue damage [88]. Several studies have indicated that iron chelators attenuate brain injury induced by ischemic stroke in animal models [89, 90] and play an antioxidant, neuroprotective role in stroke patients [91]. Recent studies have suggested that ferroptosis plays a significant role in ischemic stroke [92–94]. Ferroptosis inhibitors, ferrostatin-1 or liprostatin-1, have a protective effect on neuronal injury in middle cerebral artery occlusion (MCAO) mouse models [95]. In brain I/R mice models, the expression levels of proteins related to ferroptosis are abnormal [96]. A significant increase was demonstrated in free iron content and ROS accumulation in brain tissue, whereas the levels of GPX4 and GSH significantly decreased [97]. These studies show that ferroptosis plays a key role in brain I/R injury, and inhibition of ferroptosis could protect brain tissue damage induced by I/R.

#### 3.3.2. Target and Targeted Therapy of Ferroptosis in Cerebral I/R Injury

A deeper understanding of the target and targeted treatment of ferroptosis will be beneficial for treating related diseases. Pharmacological Se drives GPX4 expression to reduce ferroptosis, provide neuroprotection, and have a therapeutic effect on stroke [92]. A previous study found that the tau-mediated iron export pathway prevents injury induced by ferroptosis after ischemic stroke and the therapeutic potential of ferroptotic inhibition in this injury [95]. Guan et al. observed that ROS and iron levels were increased in the brains of ischemic stroke model gerbils [98]. Carvacrol exerts neuroprotective effects in cerebral ischemia by increasing the expression of GPX4 and inhibiting ferroptosis [98]. Carthamin yellow is a flavonoid isolated from safflower. In a recent study, using MCAO model rats, carthamin yellow improved cerebral I/R injury by reducing ferroptosis [96]. Galangin is a flavonoid that is mainly extracted from Chinese medicinal herbs [99]. Galangin upregulates SLC7A11 and indirectly increases the expression of GPX4, thereby inhibiting ferroptosis and attenuating cerebral I/R injury in gerbils. Galangin treatment has been shown to increase the survival rate of neurons in the brain of gerbils and alleviate learning and memory impairment after I/R [100]. It has been hypothesized that PIEZO1 participates in cerebral I/R injury by regulating ferroptosis, which might be a potential therapeutic treatment to protect against neuronal damage [101]. Furthermore, Cui et al. identified that ACSL4 is a novel neuronal death regulator, as knockout of ACSL4 could protect mice from cerebral ischemia, while forced overexpression of ACSL4 aggravated ischemic brain injury. The intervention of ACSL4 expression could be a potential therapeutic target for ischemic stroke [102]. The therapeutic targets of ferroptosis in ischemic stroke still need to be further studied to determine appropriate and more effective anti-ferroptosis-targeted therapies.

### 3.4. Ischemic Renal Injury

#### 3.4.1. Ferroptosis in Ischemic Renal Injury

Pathophysiological renal I/R injuries include renal tubular injury, inflammation, and vascular dysfunction. The precipitating factors and major early events of acute kidney injury (AKI) are renal tubular epithelial cell injury and subsequent cell death [103]. In recent years, the role of ferroptosis in renal I/R injury has been investigated. It has been suggested that ferroptosis has pathophysiological relevance in AKI [2, 103–105]. In mice with renal I/R injury, administration of ferrostatin 1 or 16–86 15 min before ischemia, renal tissue injury, and serum creatinine and urea were decreased 48 h after ischemia, indicating that ferroptosis plays an important role in the pathogenesis [106]. In mouse models of AKI induced by I/R injury, ferroptosis is directly related to the synchronous necrosis of renal tubules [106]. Ding et al. demonstrated that I/R induced upregulation of miR-182-5p and miR-378a-3p and induced ferroptosis in renal injury by downregulating GPX4 and SLC7A11 [107]. Recent studies have suggested that ferroptosis plays an important role in renal I/R injury; however, the exact mechanism of ferroptosis needs to be further studied.

#### 3.4.2. Target and Targeted Therapy of Ferroptosis in Ischemic Renal Injury

Pannexin1 (PANX1) is an ATP-releasing pathway family protein that can promote apoptosis during kidney injury. A previous study has shown that the deletion of PANX1 prevents renal I/R injury by attenuating ferroptosis activated by mitogen-activated protein kinase/extracellular signal-regulated kinase signaling [108]. Macrophage migration inhibitory factor (MIF) is a stress-regulatory cytokine. It has been reported that MIF can effectively limit necrotic ptosis, restore intracellular GSH, and reduce lipid peroxidation to alleviate oxidative stress [109]. MIF protects renal tubular epithelial cells and plays a role in renal protection during experimental I/R injury. Pachymic acid is a lanostane-type triterpenoid found in *Poria cocos*. According to research, under the treatment of pachymic acid, the renal function of mice with renal I/R injury can be improved, and renal injury can be alleviated. The protective effect of pachymic acid may be related to the inhibition of renal ferroptosis through directly or indirectly activating Nrf2 and upregulating downstream GPX4, system *x*_*c*_^−^, and HO-1 [110]. Additionally, XJB-5-131 is a mitochondrial-targeted nitroxide with a high affinity for tubular epithelial cells [111]. XJB-5-131 inhibits lipid peroxidation after I/R injury and inhibits I/R-induced ferroptosis in tubular epithelial cells, thus improving ischemic renal injury [111]. Irisin is a type of exercise-induced hormone that can ameliorate mitochondrial function and reduce ROS production. In renal I/R mice models, irisin treatment can reduce I/R-induced AKI by upregulating GPX4, a pivotal regulator of ferroptosis [112]. Inhibition of ferroptosis protects renal tubular epithelial cells and reduces renal I/R injury. These results indicate that the crucial endogenous antioxidant GPX4 and its cofactor GSH are important targets for protection against renal I/R injury.

### 3.5. Mechanism and Targeted Therapy of Ferroptosis in I/R Injury in Other Organs

Hepatic I/R, as a surgical complication after liver transplantation, can cause inflammation and immune reactions, lead to rejection, and affect prognosis [113, 114]. Galeano et al. demonstrated that subchronic, low-level iron administration has significant hepatoprotection against I/R injury [115]. A previous study has shown that ferroptosis is a mechanism of liver I/R injury in a mouse model of hepatic injury. Li et al. demonstrated that iron overload is a new risk factor for hepatic I/R injury. Ferroptosis mediates the relationship between iron overload and liver injury, showing that ferroptosis is a prospective target for preventing and treating hepatic I/R injury [116]. A recent study has also implicated that ferroptosis is associated with intestinal I/R injury [117]. Increasing evidence shows that ROS play a key role in the pathogenesis of intestinal I/R. The primary contributors of ferroptosis, including ROS generation and increase in lipid peroxidation, are related to intestinal I/R injury [118]. Deng et al. found that capsiate, a gut microbiota metabolite, can promote the expression of GPX4 by activating TRPV1 and inhibiting ferroptosis induced by intestinal I/R [119]. In addition, intestinal I/R has been shown to induce acute lung injury [120, 121]. An earlier study demonstrated ferroptosis in acute lung injury induced by intestinal I/R [122]. It has been shown that Nrf2 inhibits ferroptosis by promoting the expression of SLC7A11 and HO-1 and plays a protective role in acute lung injury following intestinal I/R [122]. The inhibitor of apoptosis-stimulating protein of p53 (iASPP) is the only inhibitor of apoptosis-stimulating protein in the p53 family. A recent study has suggested that in Nrf2-/- mice with acute lung injury induced by intestinal I/R, iASPP can effectively alleviate acute lung injury and inhibit ferroptosis through the Nrf2/HIF-1*α*/TF signaling pathway [123].

## 4. Conclusions

**Figure 3:**
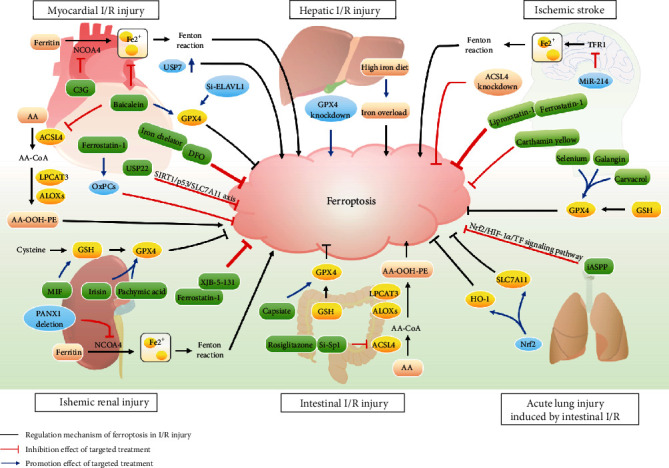
Pathological mechanism and targeted therapies of ferroptosis in ischemia-reperfusion (I/R) injury-related diseases. NCOA4: nuclear receptor coactivator 4; C3G: cyanidin-3-glucoside; USP7: ubiquitin-specific protease 7; USP7 ↑: the upregulation of USP7; ELAVL1: embryonic lethal-abnormal vision like protein 1; GPX4: glutathione peroxidase 4; AA: arachidonic acid; ACSL4: acyl-CoA synthetase long-chain family 4; LPCAT3: lysophosphatidylcholine acyltransferase 3; ALOXs: arachidonate lipoxygenases; PE: phosphatidylethanolamine; OxPCs: oxidized phosphatidylcholines; USP22: ubiquitin-specific peptidase 22; DFO: deferoxamine; TFR1: transferrin receptor 1; GSH: glutathione; MIF: macrophage migration inhibitory factor; Sp1: special protein 1; SLC7A11: solute carrier family 7 member 11; NRF2: nuclear factor erythroid 2-related factor; HO-1: heme oxygenase-1; iASPP: inhibitor of apoptosis-stimulating protein of P53.

In recent years, great progress has been made in applying ferroptosis in I/R injury in various organs and diseases (see Figure 3).  Table 1 summarizes the main findings of studies that investigated the relevant mechanisms and targets of ferroptosis in I/R injury. However, there are several unclear mechanisms of ferroptosis in I/R injury. First, existing research on the pathological effects of ferroptosis has mostly concentrated on the animal and cell levels, and evaluation of its clinical efficacy is rare. Second, there is relatively little research on the target of ferroptosis in I/R injury in various organs and the application of targeted therapy, which requires more in-depth and extensive investigations. With the in-depth exploration of research, suppressing ferroptosis could be an effective strategy for treating I/R-related organ diseases. The development of ferroptosis modulators is expected to provide new opportunities for the treatment of I/R injury and related diseases. Therefore, it is necessary to conduct such studies to screen new targeted ferroptosis therapies with potentially protective effects against I/R injury in the future.

## Figures and Tables

**Table 1:** Studies reporting the mechanisms and targets of ferroptosis in ischemia-reperfusion injury.

Ferroptosis in I/R injury	Experiment	Number (*n*)	Model/study population	Intervention	Target	Reference
Myocardial I/R injury	In vitro	Not applicable	Mouse embryonic fibroblasts	Not available	Glutaminolysis	[70]
	In vitro	Not applicable	H9c2 cells	C3G	Ferroptosis-related protein, NCOA4, RSL3	[72]
	In vitro	Not applicable	Adult rat cardiomyocytes	Ferrostatin-1	OxPCs	[76]
	In vitro	Not applicable	H9c2 cells	Baicalin	ACSL4	[82]
	In vitro	Not applicable	Human cardiomyocytes	ELAVL1 knockout	Not available	[84]
	In vivo	Not applicable	I/R-treated Sprague–Dawley rats	Not available	Upregulation of USP7	[74]
	In vivo	Not applicable	Murine models of doxorubicin- and I/R-induced cardiomyopathy	Ferrostatin-1 and iron chelator	Not available	[77]
	In vivo	Not applicable	Sprague–Dawley rats	C3G	Ferroptosis-related protein, NCOA4, RSL3	[72]
	In vivo	Not applicable	Myocardial I/R rat model	Baicalin	ACSL4	[82]
	In vivo	Not applicable	Sprague–Dawley rats	USP22	Not available	[83]
	Clinical finding	60	Patients with ST-elevation-myocardial infarct	DFO	Not available	[78]
	Clinical finding	45	Patients undergoing coronary artery bypass grafting	DFO	Not available	[79]

Cerebral I/R injury	In vitro	Not applicable	Primary cortical neurons and hippocampal HT22 cells	Selenium	GPX4	[92]
	In vivo	Not applicable	C57BL/6 mice	Selenium	GPX4	[92]
	In vivo	Not applicable	Sprague–Dawley rats	Liproxstatin-1, Ferrostatin-1	Not available	[95]
	In vivo	Not applicable	Brain I/R mice model	Carthamin yellow	Not available	[96]
	In vivo	Not applicable	Mice models of cerebral I/R	MiR-214	PVT1, TFR1, TP53	[97]
	In vivo	Not applicable	Ischemic stroke model gerbils	Carvacrol	GPX4	[98]
	In vivo	Not applicable	Ischemic stroke model gerbils	Galangin	SLC7A11, GPX4	[100]
	In vivo	Not applicable	Mice models	Knockdown of ACSL4	Not available	[102]

Ischemic renal injury	In vivo	Not applicable	C57BL/6 mice	Ferrostatin 1 or 16–86	Not available	[106]
	In vivo	Not applicable	I/R-induced renal injury rat models	Not available	GPX4, SLC7A11	[107]
	In vivo	Not applicable	Mice models	PANX1 deletion	Not available	[108]
	In vivo	Not applicable	Mif-/- mice	MIF	GSH	[109]
	In vivo	Not applicable	Renal I/R injury mice model	Pachymic acid	GPX4, SLC7A11, and HO-1	[110]
	In vivo	Not applicable	Renal I/R mice model	XJB-5-131	Not available	[111]
	In vivo	Not applicable	Renal I/R mice model	Irisin	GPX4	[112]

Hepatic I/R injury	In vivo	Not applicable	Gpx4-/- mice	Knockout of GPX4	Not available	[6]
	In vivo	Not applicable	Murine hepatic I/R injury model	Iron overload	Not available	[116]

Intestinal I/R injury	In vitro	Not applicable	Caco-2 cells	Rosiglitazone, si-special protein 1	ACSL4	[117]
	In vitro	Not applicable	Ileum organoid H/R model	Capsiate	GPX4, TRPV1	[119]
	In vivo	Not applicable	Murine model of intestinal I/R	Rosiglitazone, si-special protein 1	ACSL4	[117]
	In vivo	Not applicable	Mouse intestinal I/R model	Capsiate	GPX4, TRPV1	[119]

Acute lung injury induced by intestinal I/R	In vivo	Not applicable	C57BL/6J mice and Nrf2-/- mice	Nrf2	SLC7A11, HO-1	[122]
	In vivo	Not applicable	Nrf2-/- mice with acute lung injury induced by intestinal I/R	Inhibitor of apoptosis-stimulating protein of P53	Nrf2	[123]
